# Nationally representative estimates of health care burden of venous thromboembolism in hospitalized cancer patients

**DOI:** 10.1016/j.rpth.2026.103346

**Published:** 2026-01-13

**Authors:** Giuseppe Maiocco, Michael B. Streiff, Nareg H. Roubinian, Jacqueline Poston, Stephanie Bitner, Mohammed Hussain, Noor Khalid, Jeremy W. Jacobs, Evan M. Bloch, Aaron A.R. Tobian, Ruchika Goel

**Affiliations:** 1 Department of Internal Medicine, Mayo Clinic, Rochester, Minnesota, USA; 2 Division of Hematology/Oncology, Simmons Cancer Institute, Southern Illinois University School of Medicine, Springfield, Illinois, USA; 3 Division of Hematology, Department of Medicine, Johns Hopkins University School of Medicine, Baltimore, Maryland, USA; 4 Vitalant Research Institute, San Francisco, California, USA; 5 Division of Hematology and Oncology, Larner College of Medicine at the University of Vermont, Burlington, Vermont, USA; 6 Department of Pathology, Microbiology, and Immunology, Vanderbilt University Medical Center, Nashville, Tennessee, USA; 7 Department of Pathology, Johns Hopkins University School of Medicine, Baltimore, Maryland, USA

**Keywords:** cancer-associated thrombosis, hospitalized patients, venous thromboembolism

## Abstract

**Background:**

Venous thromboembolism (VTE) is a frequent and often preventable cancer-related comorbidity.

**Objectives:**

This study evaluated national health care burden of patients hospitalized with cancer and VTE diagnoses.

**Methods:**

The 2021 Nationwide Inpatient Sample was used to generate nationally representative estimates of cancer-associated thrombotic events identified via International Classification of Diseases-10 coding. Adjusted odds ratios (aOR) for co-diagnosis rate and all-cause mortality, median hospital charges, and length of stay (LOS) for cancer admissions with and without VTE were calculated.

**Results:**

Of 28,443,009 adult US hospitalizations in 2021, 2,907,118 (10.2%) listed cancer as a diagnosis. Of these, 234,090 (8.1%) had co-diagnosis of VTE: pulmonary embolism (PE), 96,335 (41%); deep vein thrombosis (DVT), 172,315 (74%); and PE+DVT, 34,560 (15%). Median age for admissions with cancer and VTE was 67 years (IQR, 58-76 years), with 87.6% admissions classified as major/severe underlying illness. VTE and cancer occurred at significantly higher rates than noncancer admissions (adjusted odds ratio [aOR], 1.81; 95% CI, 1.79-1.83; *P* < .001; PE: aOR, 1.54; 95% CI, 1.51-1.56; *P* < .001); DVT: aOR, 1.94; 95% CI, 1.91-1.96; *P* < .001)]. Odds of all-cause mortality for cancer admissions with VTE was higher as than without (aOR, 1.61; 95% CI, 1.56-1.67; *P* < .001; DVT: aOR, 1.41; 95% CI, 1.36-1.47; *P* < .001; PE: aOR, 1.88; 95% CI, 1.79-1.97; *P* < .001). Analysis by race found Blacks had elevated VTE odds (aOR, 1.29; 95% CI, 1.26-1.33; *P* < .001), and Blacks with VTE had elevated mortality odds (aOR, 1.15; 95% CI, 1.11-1.19; *P* < .001), compared with non-Blacks. Cancer hospitalizations with VTE were associated with significantly longer median LOS (6 days [IQR, 3-11 days] vs 4 days [IQR, 2-8 days]; *P* < .001) and higher median hospital charges ($70,416 [IQR, $36,257-$146,218] vs $55,887 [IQR, $30,276-$105,637]; *P* < .001)].

**Conclusions:**

This report on contemporary national data shows co-diagnosis of VTE and cancer is associated with significantly higher all-cause mortality, LOS, and total hospital expenditures than cancer admissions without VTE. These findings underscore importance of accurate VTE risk assessment for inpatients with cancer.

## Essentials

• Venous thromboembolism (VTE) is a frequent complication among patients with cancer.
• Co-diagnoses of VTE and cancer were evaluated via the 2021 National Inpatient Sample.
• VTE co-diagnosis was associated with 8% of cancer-associated hospitalizations.
• Patients with cancer and VTE had higher associated mortality, length of stay, and charges.

## 1 Introduction

Venous thromboembolism (VTE) (deep vein thrombosis [DVT] or pulmonary embolism [PE]) is a frequent complication and leading cause of non–cancer-related death among patients with cancer [1]. The reported frequency of cancer-associated thromboses varies considerably, with estimates between 1.6% and 8%, although risk is understood to reflect underlying patient-specific factors including cancer type, disease stage, specific treatments, and comorbidities [[2], [3], [4], [5], [6]]. Preceding studies focused specifically on in-patients with cancer reported VTE events in 4.1% of hospitalizations, with DVT in 3.4% and PE in 1.1% [6,7]. Rates of VTE further differ by specific cancers, with increased frequency in solid tumors, including in cancers of the pancreas (8.1%), kidney (5.6%), ovary (5.6%), lung (5.1%), and stomach (4.9%) [6]. VTE incidence in hematologic malignancies is less frequent compared with that in solid tumors, although elevated rates have been described in myeloma (5.0%), non-Hodgkin lymphoma (4.8%), and Hodgkin lymphoma (4.6%) [6]. Concurrent anticancer treatment has been shown to further elevate VTE risk [7]. When compared with the hospitalized patients without cancer, a study demonstrated a 4-fold elevation in VTE risk in cancer patients and greater than 6-fold with concurrent chemotherapy [7]. These findings, however, are not static; over the past several decades, increasing VTE rates in patients with cancer have been observed [2,6].

Cancer-associated thromboses represent a significant source of inpatient mortality and health care expenditure [[8], [9], [10], [11], [12]]. Fatal PE has been demonstrated to be 3 times more likely in patients with cancer than those without [13,14]. In a hospitalized cancer population comparing patients with VTE vs those without VTE events, the 30-day case fatality rate was shown to be significantly elevated (19.1% vs 3.6%) [14]. Case fatality rates were further shown to differ between PE and DVT, with elevated rates comparatively in PE of 9.7% vs 4.6% in DVT. Similarly, among hospitalized neutropenic cancer patients, those with VTE had an elevated in-hospital mortality rate [9]. Cancer-associated VTE events are additionally associated with significant increase in hospitalized health care expenditures [11,12]. Prior medical claims-based cost analyses have demonstrated that cancer patients with VTE consume significantly more health care resources than cancer patients without VTE [11,12].

While studies from smaller cohorts have described the associated risk and mortality with VTE and cancer, significant variability in these reported data remains, compounded by disparate rates among cancer subtypes. In addition, at present, the national health care burden of cancer-associated thromboembolic events and its effect on clinical outcomes on these patients has not been investigated. This, compounded with the increasing VTE incidence over preceding decades and rapidly evolving cancer treatment landscape, underscores the need for contemporary estimates of cancer-associated thrombosis [2,6]. This study seeks to use a nationally representative database to report the contemporary rates of co-diagnosis, mortality, and health care burden of cancer and VTE among patients hospitalized in the United States.

## 2 Methods

The Nationwide Inpatient Sample Healthcare Cost and Utilization Project (HCUP-NIS) is the largest all-payer inpatient database in the United States, approximating a 20% stratified sample of inpatient discharges from >5000 hospitals across 48 states. This sampling methodology is estimated to cover 97% of the US population. Within the NIS, the unit of analysis (observation) is a hospital discharge, not a specific patient; thus, it is possible for a patient with multiple hospitalizations during 2021 to be captured each time as a separate hospitalization. Observations are self-weighted, with NIS-provided discharge weights used to generate nationally representative estimates, defined by census division, bed size, location, teaching status, and hospital ownership [15].

All NIS data are patient and hospital de-identified. These data include 1 primary or principal diagnosis and up to 39 secondary diagnosis codes, 1 primary and up to 24 secondary procedure codes, admission and discharge status, demographic information, and hospital characteristics. The principal diagnosis is the primary reason for hospital admission. All listed diagnoses and procedures are defined via International Classification of Diseases, Tenth Revision, Clinical Modification (ICD-10-CM) or Procedure Coding System (ICD-10-PCS) coding.

Within the NIS family of databases, the Clinical Classifications Software Refined (CCSR) database aggregates ICD-10-CM/PCS codes into clinically meaningful categories [16]. The CCSR database aggregates >70,000 ICD-10-CM diagnosis codes into >530 clinical categories across 22 body systems and >80,000 ICD-10-PCS procedure codes into >320 clinical categories across 31 clinical domains, respectively. In addition to creation of clinically specific categories, CCSR designations delineate among primary or principal diagnosis only, secondary diagnosis only, and both primary and secondary diagnosis.

Data from the 2021 HCUP-NIS were used to generate nationally representative estimates of cancer-associated venous thrombotic events; CCSR codes were used to identify hospital discharges in which any diagnosis (primary and/or secondary) of cancer (CCSR NEO001-NEO072) or VTE (CCSR CIR013 [acute pulmonary embolism], CIR033 [acute phlebitis; thrombophlebitis and thromboembolism], CIR034 [chronic phlebitis; thrombophlebitis and thromboembolism]) were listed. Sensitivity testing were performed regarding inclusion of NEO072 (neoplasms of unspecified nature or uncertain behavior (Supplementary Tables 2 and 3) and impact on co-diagnosis rate and mortality. Inclusion vs exclusion did not result in changes to the conclusions of this study. Other myeloproliferative disorders, for example, polycythemia vera, essential thrombocythemia, or myelofibrosis, were not included in the present analysis. Analysis was limited to hospitalizations 18 years of age and older. Given mid-pandemic database timing, cancer and VTE hospitalizations with COVID-19 diagnosis were identified in 5.7% of our sample. Due to low propensity, this population was not excluded from our analysis.

The NIS includes demographics (age, sex, and race) and hospital-level characteristics including location (urban vs rural), teaching status, bed size, admission/discharge status, total charges, expected payment source, and hospitalization length. The variable race includes both race and ethnicity as a single, mutually exclusive category per discharge record, collected at the hospital level, and may either be self-reported or observational. Specific categories include White, Black, Hispanic, Asian/Pacific Islander, Native American, and other. Additionally, HCUP-NIS supplies an All Patients Refined Diagnostic Related Groups (APRDRG) rating, a validated inpatient clinical severity classification system widely used in the US as a case-mix measure, accounting for illness severity, mortality risk, prognosis, treatment difficulty, need for intervention, and resource intensity. Importantly, data regarding laboratory values and specific pharmacological therapies used during an admission are not included within the HCUP dataset.

The NIS is a de-identified, publicly available data set. Therefore, the study was deemed exempt from review by the Southern Illinois University School of Medicine institutional review board. This analysis was conducted following HCUP data use agreement guidelines, including suppression of tabulated data values between 10 and 1 due to risk of disclosure [17].

Data analysis was performed via STATA V18.0 (Statacorp) using survey analysis commands applying the sampling weights as determined by HCUP. Demographic and clinical characteristics were described as counts, percentages, mean (SDs), and median (IQR) as appropriate. Odds ratios (ORs) were calculated, and statistical comparisons of proportions and medians were performed as applicable. Adjusted odds ratios (aORs) were included for applicable analyses. For comparison between cancer and noncancer hospitalizations, ORs were adjusted for age, sex, comorbidities (obesity, type 2 diabetes mellitus, hypertension, tobacco use, and sepsis), and APRDRG severity index. For comparison between cancer hospitalizations with or without thrombus co-diagnosis, ORs were adjusted for age, sex, aforementioned comorbidities and presence of metastatic disease for assessment of disease stage; APRDRG severity index was not used given collinearity due to near ubiquitous elevated severity ratings in cancer/VTE hospitalization group. All *P* values were 2-tailed with statistical significance set at *P* < .05. Statistical analyses performed includes the Mann–Whitney U-test for hospital length of stay and total costs and Wald chi-squared test for comparison of rates of co-diagnosis and all-cause mortality. Cost analyses were collected from NIS data, which report as hospital charges, not including professional fees or noncovered charges. Overall, data missing were small and consistent between analyzed groups. Numerically, these included <3% for race and <0.2% for sex, severity, and payer information. Missing data were not present regarding teaching status of hospital, hospital bed size, hospital region, or length of stay and charge data. We used the STROBE cross-sectional checklist when writing our report [18].

## 3 Results

Of 28,443,009 adult US hospitalizations in 2021, 2,907,118 (10.2%) involved patients with cancer as any listed diagnosis (Table 1). Of all cancer-related hospitalizations, 234,090 (8.1%) included a co-diagnosis of VTE. Of these hospitalizations, 16.7% (39,190/234,090) listed VTE as the primary hospital diagnosis. The cancer VTE cohort included 41% (96,335) with a PE diagnosis and 74% (172,315) with a DVT diagnosis. This represents a 15% (34,560) overlap with diagnoses of both PE and DVT. Median age for patients hospitalized with cancer and VTE diagnoses was 67 years (IQR, 58-76 years); 51.2% were males and 65.6% were Whites.

**Table 1:** Data sample characteristics for all Nationwide Inpatient Sample (NIS) hospitalizations in 2021, for all cancer-related hospitalizations and cancer-related hospitalizations with VTE.

Data sample characteristics^a^	All hospitalizations	Any cancer diagnosis	Cancer and VTE diagnoses
Population size	28,443,009	2,907,118	234,090
Age (y)	61 (40-74)	68 (59-77)	67 (58-76)
Sex			
Female	15,951,656 (56.1)	1,379,078 (47.4)	114,050 (48.7)
Male	12,481,238 (43.9)	1,527,274 (52.5)	119,970 (51.2)
Race			
White	18,113,030 (65.4)	2,016,048 (69.3)	153,470 (65.6)
Black	4,332,739 (15.6)	377,170 (13.0)	38,030 (16.2)
Hispanic	3,434,117 (12.4)	262,465 (9.0)	22,510 (9.6)
Other	1,807,578 (6.5)	184,160 (6.3)	14,530 (6.2)
APRDRG rated disease severity			
Minor or moderate	16,860,403 (59.3)	1,214,959 (41.8)	28,990 (12.4)
Major or severe	11,558,501 (40.6)	1,692,054 (58.2)	205,090 (87.6)
Insurance			
Medicare	13,067,733 (45.9)	1,722,913 (59.3)	132,105 (56.4)
Medicaid	5,473,193 (19.2)	310,115 (10.7)	28,210 (12.1)
Private	7,678,592 (27.0)	721,465 (24.8)	61,385 (26.2)
Self-pay	1,157,973 (4.1)	59,610 (2.1)	5305 (2.3)
Other	1,019,528 (3.6)	89,085 (3.1)	6850 (2.9)
Teaching status of hospital			
Teaching hospital	21,034,803 (74.0)	2,314,115 (79.6)	192,820 (82.4)
Nonteaching	7,408,205 (26.0)	593,003 (20.4)	41,270 (17.6)
Hospital bed size			
Large	13,956,486 (49.5)	1,633,185 (56.2)	137,795 (58.9)
Small	14,486,522 (50.9)	1,273,933 (43.8)	96,295 (41.1)
Hospital region			
Northeast	5,215,304 (18.3)	599,834 (20.6)	47,810 (20.4)
Midwest	6,210,981 (21.8)	656,226 (22.6)	53,255 (22.7)
South	11,502,015 (40.4)	1,093,599 (37.6)	86,550 (37.0)
West	5,514,709 (19.4)	557,429 (19.2)	46,475 (19.9)
COVID-19	2,417,760 (8.5)	131,325 (4.5)	13,250 (5.7)
Length of stay (d)	3 (2-6)	4 (2-8)	6 (3-12)
Hospital charges ($)	39,691 (21,127-78,135)	55,887 (30,276-105,637)	70,416 (36,257-146,218)

Values are median (IQR) or *n* (%).

APRDRG, All Patients Refined Diagnostic Related Groups; VTE, venous thromboembolism.

a Missing data in race include 2.7%, 2.3%, and 2.4% for all hospitalizations, any cancer diagnosis, and cancer and VTE diagnoses, respectively. Missing data were <0.2% for sex, APRDRG rated disease severity, and insurance. There were no missing data for age, teaching status, hospital bed size, hospital region, length of stay, and hospital charges.

### 3.1 Hospitalization characteristics

Among patients hospitalized with concomitant cancer and VTE diagnoses, 87.6% were classified as having major/severe underlying illness as rated by APRDRG mortality risk stratification (Table 1). Hospitalizations predominantly occurred in teaching hospitals (82.4%), with 58.9% large vs 41.1% small bed. US hospitalization distribution included the South (37.0%), Midwest (22.7%), Northeast (20.4%), and West (19.9%). A COVID-19 co-diagnosis was present in 5.7% of the cancer/VTE cohort compared with 8.5% of the overall NIS population.

### 3.2 Cancer and thrombosis co-diagnosis

The aORs of VTE in cancer admissions compared with that in noncancer admissions was 1.81 (95% CI, 1.79-1.83; *P* < .001) (Table 2). aORs of PE was 1.54 (95% CI, 1.51-1.56; *P* < .001), and DVT was 1.94 (95% CI, 1.91-1.96; *P* < .001). Compared with hospitalizations of non-Black patients, aORs of VTE in hospitalizations of Black patients was 1.29 (95% CI, 1.26-1.33; *P* < .001). Using CCSR groupings of ICD-10 coding, VTE diagnosis was further evaluated by specific cancer groupings (Supplementary Table 1). VTE was most frequently co-diagnosed in liver (17.5%), pancreatic (15.1%), bile duct (14.8%), adrenocortical (12.2%), testicular (11.7%), gallbladder (11.5%), uterine (11.5%), and endometrial (11.4%) cancer admissions (Table 3). Among hematologic malignancies, the highest rates of co-diagnosis were found in acute myeloid leukemia (8.1%), hairy cell leukemia (7.8%), non-Hodgkin lymphoma (7.8%), Hodgkin lymphoma (7.5%), and acute lymphoblastic leukemia (6.9%). In our cancer cohort, PE and DVT were further analyzed by clot location (Table 3). PE were most commonly diagnosed segmental (75.6%), 19.9% subsegmental, and 4.7% saddle emboli. DVT were predominantly diagnosed in the lower extremities (55.1%), with 31.4% proximal and 18.0% distal. DVT burden was also identified in upper extremity (19.0%), portal (13.1%), jugular (5.9%) and thoracic (5.9%) veins.

**Table 2:** Odds ratio of venous thromboembolism in hospitalizations in patients with or without cancer diagnosis.

	Thromboembolus diagnosis		Unadjusted			Adjusted		
	Cancer present (%)	Cancer absent (%)	OR^a^	95% CI	*P*	aOR^b^	95% CI	*P*
VTE	8.1	3.5	2.43	2.41-2.46	<.001	1.81	1.79-1.83	<.001
PE	3.3	1.6	2.05	2.02-2.08	<.001	1.54	1.51-1.56	<.001
DVT	5.9	2.3	2.62	2.59-2.66	<.001	1.94	1.91-1.96	<.001

aOR, adjusted odds ratio; DVT, deep vein thrombosis; OR, odds ratio; PE, pulmonary embolism; VTE, venous thromboembolism.

a Unadjusted OR of specified thrombosis with or without a cancer diagnosis.

b Age, sex, comorbidity (hypertension, type 2 diabetes mellitus, tobacco use, obesity, and sepsis), and APRDRG disease severity aOR.

**Table 3:** Inpatient venous thromboembolism by cancer type and thrombosis location.

Cancer subtype	Count	VTE	%	Thrombosis location	Count	%
Liver	63,750	11,135	17.5	Pulmonary embolism^a^	96,335	
Pancreatic	114,565	17,330	15.1	Segmental	72,835	75.6
Bile duct	26,435	3905	14.8	Subsegmental	19,215	19.9
Adrenocortical	740	90	12.2	Saddle	4525	4.7
Testicular	7680	900	11.7	Septic	1015	1.1
Gallbladder	7800	900	11.5			
Uterine	14,340	1645	11.5	Deep vein thrombosis^a^	172,315	
Endometrial	38,340	4355	11.4	Lower extremity	94,995	55.1
Stomach	46,895	4970	10.6	Proximal	54,140	31.4
Urethral	2025	200	9.9	Distal	30,980	18.0
Cervical	27,550	2650	9.6	Upper extremity	32,715	19.0
Cardiac	2985	285	9.5	Portal vein	22,640	13.1
Parathyroid	265	25	9.4	Jugular	10,210	5.9
Ovarian	57,040	5375	9.4	Thoracic	10,200	5.9
Sarcoma	26,665	2465	9.2	Renal	3235	1.9
Small intestinal	12,215	1115	9.1	Central venous sinus thrombosis	155	0.1
Respiratory	384,635	34,565	9.0			

a Individual diagnoses; individual hospitalizations may contain diagnoses of multiple co-diagnosed thromboemboli.

### 3.3 Mortality

Rate of all-cause mortality in all cancer–related hospitalizations compared with that of all non–cancer-related hospitalizations irrespective of VTE diagnosis was 6.1% vs 3.5% (aOR, 1.29; 95% CI, 1.27-1.31; *P* < .001). Among the cancer-related hospitalizations, admissions with VTE diagnosis had higher aORs of all-cause mortality than those without VTE diagnosis (aOR, 1.61; 95% CI: 1.56-1.67; *P* < .001) (Table 4). Specifically, hospitalizations of Black patients with cancer and VTE diagnoses had higher all-cause mortality (aOR, 1.15; 95% CI, 1.11-1.19; *P* < .001) than hospitalization of non-Black patients with cancer and VTE diagnoses. By clot type, aORs of all-cause mortality in cancer hospitalizations in DVT was 1.41 (95% CI, 1.36-1.47; *P* < .001) and in PE 1.88 (95% CI, 1.79-1.97; *P* < .001).

**Table 4:** Odds ratio of mortality in cancer-associated hospitalizations with or without venous thromboembolism diagnosis.

	All-cause mortality		Unadjusted			Adjusted		
	With thrombus	Without thrombus	OR^a^	95% CI	*P*	aOR^b^	95% CI	*P*
VTE	10.5	5.7	1.93	1.87-1.99	<.001	1.61	1.56-1.67	<.001
PE	11.7	5.9	2.11	2.02-2.21	<.001	1.88	1.79-1.97	<.001
DVT	9.7	5.9	1.72	1.66-1.79	<.001	1.41	1.36-1.47	<.001

aOR, adjusted odds ratio; DVT, deep vein thrombosis; OR, odds ratio; PE, pulmonary embolism; VTE, venous thromboembolism.

a Unadjusted OR of mortality in hospitalizations with cancer and specified thrombosis diagnoses.

b Age, sex, comorbidity (hypertension, type 2 diabetes mellitus, tobacco use, obesity, and sepsis), and metastatic disease aOR.

### 3.4 Co-diagnoses/procedures

The most frequent co-diagnoses in patients hospitalized with cancer and VTE diagnoses included hypertension (59.4%), secondary metastasis (46.0%), anemia (38.6%), dyslipidemia (35.3%), and respiratory failure (30.2%) (Table 5); 17.1% of this cohort carried at least 1 diagnosis, which may have represented a contraindication for anticoagulation including 12.7% with thrombocytopenia and 5.2% with gastrointestinal bleed. Most frequent coded procedural interventions in cancer and VTE-related hospitalizations were red blood cells or platelet transfusion (14.4%), respiratory ventilation (11.8%), endoscopy/colonoscopy (8.9%), thoracentesis (7.6%), and airway insertion (7.3%).

**Table 5:** Cancer and venous thromboembolism co-diagnoses and procedures (cancer and venous thromboembolism admissions, *n* = 234,090).

Co-diagnoses	Count	%	Procedures	Count	%
Hypertension	139,090	59.4	RBC/platelet transfusion	33,760	14.4
Secondary metastasis	107,715	46.0	Respiratory ventilation	27,540	11.8
Anemia	90,455	38.6	Endoscopy/colonoscopy	20,765	8.9
Dyslipidemia	82,645	35.3	Thoracentesis	17,895	7.6
Respiratory failure	70,695	30.2	Airway insertion	17,200	7.3
Renal failure	69,540	29.7	Paracentesis	15,665	6.7
Type 2 diabetes mellitus	60,580	25.9	IVC filter	14,670	6.3
Malnutrition	60,580	25.9	Antineoplastic therapy	12,505	5.3
Sepsis	48,120	20.6	Mechanical thrombectomy	8,315	3.6
Hyponatremia	47,710	20.4	Bronchoscopy	8,140	3.5

IVC, inferior vena cava; RBC, red blood cell.

### 3.5 Health care burden

Among cancer hospitalizations with concomitant VTE diagnosis median length of stay was 6 days (IQR, 3-12 days) vs 4 days (IQR, 2-8 days) compared with cancer hospitalizations without VTE (*P* < .001). Hospital charges in cancer hospitalizations with vs without VTE were $70,416 (IQR, $36257-$146,218) vs $55,887 (IQR, $30,276-$105,637; *P* < .001).

## 4 Discussion

This study uses a nationally representative inpatient database to evaluate the rate, mortality, and health care burden of co-diagnosis of VTE and cancer in hospitalized patients in the United States. In this study, approximately 8% of nearly 3 million cancer hospitalizations reported a co-diagnosis of VTE, with more than double the aORs than that of noncancer hospitalizations. Both PE and DVT were associated with a significantly higher aORs of diagnosis in cancer-related admissions than those with in non–cancer-related admissions. Cancer hospitalizations involving Black patients demonstrated a 30% increased aORs of VTE diagnosis than generalized hospitalizations. We interpret this rate of co-diagnosis cautiously in comparison with prior studies that evaluated true incidence; evaluating hospitalized cancer patients, Khorana et al. [6] reported VTE incidence in hospitalized cancer patients at 4.1%, with DVT at 3.4% and PE at 1.1%. There is mixed evidence suggesting rising VTE incidence in cancer patients. In a hospitalized cancer cohort, VTE rates reportedly increased from 3.5% in 1995 to 6.5% in 2012 [19]. Cancer-associated thrombosis increased from 2005-2007 to 2014-2017 in a California cohort, whereas a Danish cohort demonstrated a threefold increase in cancer-related VTE risk over 20 years and a 6-fold increase among those on chemotherapy or targeted therapies [2,5]. In contrast, no significant trend was observed following a Veterans affairs cancer cohort from 2006 and 2021 [20]. Observed rates of VTE diagnosis in cancer hospitalizations likely include effects of therapeutic advances and comorbidities but should also interpreted with consideration to alternative explanations including improved diagnosis capture in the form of electronic health record advancement.

Thromboembolic burden is further understood to vary by cancer subtype. Using CCSR groupings of ICD-10 coding, this study was uniquely able to evaluate >60 specific cancers and associated rate of co-diagnosed VTE on a national scale. Consistent with preceding studies, the highest rates of thrombosis were observed in gastrointestinal (liver, pancreatic, and biliary) and genitourinary (uterine, testicular, and endometrial) malignancies. Notably, the co-diagnosis of VTE in patients with brain cancers, while elevated, was lower than previously reported [21]. This finding is likely due in part to the spectrum of brain cancers included within ICD-10 coding. Hematologic malignancies including acute leukemia, lymphoma, and multiple myeloma have also been associated with elevated thrombotic risk, as corroborated in this study [22]. However, the rate of hematological cancer thrombosis was observed to be lower than that of solid tumors. Differences in the rate of thrombosis in hematological malignancies may be partly explained via treatment associated risks including thrombocytopenia and concurrent bleeding, which may affect implementation of thromboprophylaxis [23].

Cancer-associated thromboses are a major cause of non–cancer-related death. Significantly, our analysis demonstrated a 60% elevation in the aORs of all-cause mortality in patients hospitalized with a cancer and VTE diagnosis vs without VTE diagnosis. The ORs for mortality increased to nearly double for hospitalizations with PE and above 40% for those with DVT. Additionally, hospitalizations of Black patients with cancer and VTE had a nearly 15% increased aORs of all-cause mortality compared with general hospitalizations with cancer and VTE. In total, these data represent a significant elevation in attributable risk for an already at-risk hospitalized cancer population. These findings are consistent with previous reports: Lyman et al. [19] observed in-hospital mortality in 15.0% of cancer patients with VTE compared with 5.5% without VTE; this rate increased to 19.4% for patients with PE specifically [19]. A cross-sectional NIS analysis evaluating hospitalizations with metastatic cancer with vs without VTE diagnosis between 2008 and 2013 found an OR of in-hospital mortality of 1.50 [24]. Notably, a large subset of our current analysis (46.0%) possessed a diagnosis of metastatic cancer. Increased disease stage is known to confer an elevated thrombotic risk [3,[25], [26], [27]]. Disease status of our patient cohort was further contextualized by associated comorbidities and procedural utilization. Hospitalizations with both cancer and VTE diagnoses possessed comorbidities associated with both chronic disease processes, such as hyperlipidemia and hypertension, as well as acute disease processes (eg, renal failure). Procedurally, these hospitalizations most frequently used blood product transfusions, including RBC and platelets, while more than 5% received in hospital chemotherapy. The transfusions could be attributed to the primary underlying disease, chemotherapy, and/or bleeding, which could be related/unrelated to anticoagulation.

Cancer-related hospitalizations with VTE co-diagnosis incurred higher cost, median hospital expenditure, and median length of stay than cancer hospitalizations without diagnosed VTE. Hospital expenditure data presented in this study are elevated although proportional in comparison with a 2016 Minnesotan study that found adjusted mean predicted costs were 1.9-fold higher for cancer patients with VTE than that for controls ($49,351 vs $26,529) over a 5-year time frame [28]. Likewise, an analysis using an Israeli national cancer registry found that, between 2010 and 2018, a VTE subcohort was more likely to be hospitalized, have longer inpatient stays, have an emergency room visit, and increased primary care physician visits than matched controls [29]. Another study showed cancer patients with VTE incurred 3 times as many hospitalizations, increased days in hospital (10.19 vs 3.37), and increased total health care costs ($74,959 vs $41,691) [11].

Management of cancer-associated venous thromboembolic disease for both ambulatory and inpatient disease has been evolving. Most recently, updated National Comprehensive Cancer Network guidelines for cancer-associated venous thromboembolic disease were published in 2024 [30]. Ambulatory prophylaxis has been shown to significantly reduce VTE incidence in cancer patients receiving chemotherapy, with a 2020 meta-analysis evaluating VTE with any versus no prophylaxis demonstrating a 49% VTE risk reduction without elevated bleeding risk [31]. National Comprehensive Cancer Network recommendations for outpatient prophylactic anticoagulation in patients at intermediate to high risk for VTE (Khorana score ≥ 2) are with direct oral anticoagulants or subcutaneous low-molecular-weight heparin [30]. Inpatient medical prophylaxis regimens include low-molecular-weight heparin, fondaparinux or unfractionated heparin. Prophylaxis is often complicated by clinical patient factors, such as bleeding and thrombocytopenia. However, multiple previous studies have demonstrated underuse of prophylactic regimens in hospitalized cancer inpatients to date [[32], [33], [34], [35]]. Further, previous data have estimated approximately 10% to 20% of hospital acquired VTE in cancer patients are preventable [[35], [36], [37], [38]]. Thromboembolic prophylaxis in hospitalized cancer patients remains a key prevention strategy, which could improve patient outcomes and reduce cancer-associated health care expenditure.

This study has several limitations. First, the NIS data reflect a rate of co-diagnosis, capturing hospitalizations in which both cancer and VTE diagnoses were present. This could be new cases diagnosed during hospitalization or a previously diagnosed case of VTE. Because VTE temporality cannot be identified, these data likely encompass cancer patients with both VTE developed in the outpatient setting resulting in subsequent hospital admission, as well as VTE events occurring during hospitalization. Second, the analysis cannot define direct attribution of thrombus to cancer; although aiming to control for disease severity or disease stage, thrombosis occurrence unrelated to patient’s cancer directly, such as concurrent to procedure, cannot be excluded. This analysis includes data during the COVID-19 pandemic. Although low rates of COVID co-diagnosis in our cohort are reassuring given additional prothrombotic risk, this may also signify that fewer low risk cancer patients were hospitalized. COVID-19 additionally may have delayed cancer treatments, potentially impacting VTE risk. Third, the NIS’s large sample size enables the analysis of infrequent diagnoses; however, this also introduces potential biases. As the NIS records each hospitalization as an independent event, patients with multiple admissions may be counted multiple times. This risk is particularly relevant for rare diseases, although the observed diverse geographic distribution implies possible mitigation of this limitation. Fourth, the accuracy of our findings from an administrative database is contingent on coding reliability at time of billing; carryover diagnoses from prior hospitalizations may potentially inflate diagnosis counts, whereas under documentation may lead to underrepresentation. Recent studies have sought to evaluate validity of ICD-10 coding for VTE, including in both cancer and noncancer populations, demonstrating a positive predictive value ranging between 76% and 95% and sensitivity of 58% and 68% for principal discharge codes [[39], [40], [41]]. Inclusion of secondary discharge codes was found to improve sensitivity at the expense of positive predictive value, a consideration in our decision to include chronic thromboembolism and secondary thrombosis diagnosis in this evaluation. Bikdeli et al. [39] further details a composite methodology using additional imaging codes to optimize case identification [41], which was not applied in this study but may inform future work. Fifth, because the NIS only captures all-cause mortality, specific causes of death cannot be determined, limiting ability to directly attribute mortality to VTE-related complications within this cohort. Although we adjusted for age, sex, severity, and specific comorbidities, there remains unadjusted variables represent an additional source of confound. With health care expenses, NIS quotes hospital charges, which represent the total amount a hospital bills for a patient’s care, while cost reflects the actual expenses incurred by the hospital to provide that care, including wages, supplies, and utilities. Although evaluating hospitalizations with diagnoses of cancer and thrombosis, attribution of a specific portion of these charges to VTE is not possible. Finally, prophylactic anticoagulation both prior to admission and in-hospital is unable to be assessed via the NIS, which would be useful in determining increased regimen adherence vs event burden while on anticoagulation.

## 5 Conclusion

In conclusion, these nationally representative data show that VTE is a frequent co-diagnosis among hospitalized patients with cancer. The most common cancers co-diagnosed with VTE include gastrointestinal and genitourinary cancers, as well as those with metastases. Patients hospitalized with diagnoses of cancer and VTE were associated with significantly higher all-cause mortality, length of hospitalization, and total hospital expenditures than patients with cancer without VTE. Hospitalizations of Black patients with cancer diagnoses had an elevated OR of VTE diagnosis, and those with VTE diagnosis had higher all-cause mortality than other races. These findings capture the health care burden of cancer-associated venous thrombosis in inpatients nationally and highlight the opportunities for optimization of VTE management in cancer patients.
