# Supplementary material for: Nationally representative estimates of health care burden of venous thromboembolism in hospitalized cancer patients
Source: Res Pract Thromb Haemost. 2026 Jan 13;10(1):103346. doi: 10.1016/j.rpth.2026.103346 (PMC12914204; doi:10.1016/j.rpth.2026.103346)
Supplement: Supplementary Tables 1-3 [file mmc1.docx]

**Supplementary Table 1: Rates of venous thromboembolism, pulmonary embolism, and deep vein thrombosis diagnoses among cancer subgroups**

|  |  | **Total** | **VTE** |  | **PE** |  | **DVT** |  |
| --- | --- | --- | --- | --- | --- | --- | --- | --- |
|  | **General Adult Population** | **28,443,009** | **1,121,864** | **3.9%** | **515,859** | **1.8%** | **770,929** | **2.7%** |
|  | **All Cancer Average** | **2,907,118** | **234,090** | **8.1%** | **96,335** | **3.3%** | **172,315** | **5.9%** |
| NEO017 | Gastrointestinal cancers – liver | 63,750 | 11,135 | 17.5% | 1,410 | 2.2% | 10,360 | 16.3% |
| NEO051 | Endocrine system cancers - pancreas | 114,565 | 17,330 | 15.1% | 6,895 | 6.0% | 13,680 | 11.9% |
| NEO018 | Gastrointestinal cancers - bile duct | 26,435 | 3,905 | 14.8% | 1,120 | 4.2% | 3,220 | 12.2% |
| NEO053 | Endocrine system cancers - adrenocortical | 740 | 90 | 12.2% | 40 | 5.4% | 75 | 10.1% |
| NEO069 | Cancer of other sites | 6,740 | 800 | 11.9% | 375 | 5.6% | 595 | 8.8% |
| NEO040 | Male reproductive system cancers - testis | 7,680 | 900 | 11.7% | 340 | 4.4% | 705 | 9.2% |
| NEO019 | Gastrointestinal cancers - gallbladder | 7,800 | 900 | 11.5% | 450 | 5.8% | 645 | 8.3% |
| NEO031 | Female reproductive system cancers - uterus | 14,340 | 1,645 | 11.5% | 735 | 5.1% | 1,215 | 8.5% |
| NEO035 | Female reproductive system cancers - endometrium | 38,340 | 4,355 | 11.4% | 2,150 | 5.6% | 3,045 | 7.9% |
| NEO071 | Malignant neoplasm, unspecified | 33,885 | 3,735 | 11.0% | 1,865 | 5.5% | 2,525 | 7.5% |
| NEO070 | Secondary malignancies | 978,259 | 107,715 | 11.0% | 42,870 | 4.4% | 77,779 | 8.0% |
| NEO013 | Gastrointestinal cancers - stomach | 46,895 | 4,970 | 10.6% | 2,295 | 4.9% | 3,615 | 7.7% |
| NEO046 | Urinary system cancers - urethra | 2,025 | 200 | 9.9% | 70 | 3.5% | 150 | 7.4% |
| NEO032 | Female reproductive system cancers - cervix | 27,550 | 2,650 | 9.6% | 775 | 2.8% | 2,180 | 7.9% |
| NEO011 | Cardiac cancers | 2,985 | 285 | 9.5% | 125 | 4.2% | 200 | 6.7% |
| NEO038 | Female reproductive system cancers - all other types | 3,370 | 320 | 9.5% | 165 | 4.9% | 230 | 6.8% |
| NEO054 | Endocrine system cancers - parathyroid | 265 | 25 | 9.4% | 5 | 1.9% | 25 | 9.4% |
| NEO033 | Female reproductive system cancers - ovary | 57,040 | 5,375 | 9.4% | 2,635 | 4.6% | 3,845 | 6.7% |
| NEO024 | Sarcoma | 26,665 | 2,465 | 9.2% | 725 | 2.7% | 1,965 | 7.4% |
| NEO021 | Gastrointestinal cancers - all other types | 22,350 | 2,055 | 9.2% | 680 | 3.0% | 1,695 | 7.6% |
| NEO014 | Gastrointestinal cancers - small intestine | 12,215 | 1,115 | 9.1% | 415 | 3.4% | 910 | 7.4% |
| NEO022 | Respiratory cancers | 384,635 | 34,565 | 9.0% | 20,180 | 5.3% | 20,395 | 5.3% |
| NEO012 | Gastrointestinal cancers - esophagus | 41,100 | 3,685 | 9.0% | 1,765 | 4.3% | 2,545 | 6.2% |
| NEO045 | Urinary system cancers - kidney | 90,740 | 8,045 | 8.9% | 2,595 | 2.9% | 6,455 | 7.1% |
| NEO067 | Mesothelioma | 6,685 | 585 | 8.8% | 290 | 4.3% | 410 | 6.1% |
| NEO042 | Male reproductive system cancers - all other types | 540 | 45 | 8.3% | 20 | 3.7% | 30 | 5.6% |
| NEO016 | Gastrointestinal cancers - anus | 10,910 | 895 | 8.2% | 295 | 2.7% | 725 | 6.6% |
| NEO020 | Gastrointestinal cancers - peritoneum | 4,820 | 395 | 8.2% | 170 | 3.5% | 300 | 6.2% |
| NEO060 | Leukemia - acute myeloid leukemia (AML) | 70,605 | 5,710 | 8.1% | 1,270 | 1.8% | 4,820 | 6.8% |
| NEO047 | Urinary system cancers - all other types | 3,470 | 275 | 7.9% | 100 | 2.9% | 205 | 5.9% |
| NEO063 | Leukemia - hairy cell | 2,805 | 220 | 7.8% | 85 | 3.0% | 170 | 6.1% |
| NEO048 | Nervous system cancers - brain | 53,475 | 4,180 | 7.8% | 2,195 | 4.1% | 3,130 | 5.9% |
| NEO058 | Non-Hodgkin lymphoma | 205,480 | 15,950 | 7.8% | 5,240 | 2.6% | 12,535 | 6.1% |
| NEO041 | Male reproductive system cancers - penis | 2,280 | 175 | 7.7% | 65 | 2.9% | 140 | 6.1% |
| NEO056 | Endocrine system cancers - all other types | 2,435 | 185 | 7.6% | 80 | 3.3% | 140 | 5.7% |
| NEO030 | Breast cancer - all other types | 164,705 | 12,470 | 7.6% | 6,120 | 3.7% | 8,270 | 5.0% |
| NEO057 | Hodgkin lymphoma | 16,560 | 1,250 | 7.5% | 450 | 2.7% | 945 | 5.7% |
| NEO066 | Malignant neuroendocrine tumors | 37,785 | 2,795 | 7.4% | 990 | 2.6% | 2,130 | 5.6% |
| NEO049 | Nervous system cancers - all other types | 2,840 | 205 | 7.2% | 90 | 3.2% | 150 | 5.3% |
| NEO023 | Bone cancer | 17,525 | 1,240 | 7.1% | 465 | 2.7% | 900 | 5.1% |
| NEO059 | Leukemia - acute lymphoblastic leukemia (ALL) | 27,205 | 1,890 | 6.9% | 405 | 1.5% | 1,640 | 6.0% |
| NEO036 | Female reproductive system cancers - vulva | 6,340 | 440 | 6.9% | 145 | 2.3% | 335 | 5.3% |
| NEO052 | Endocrine system cancers - thymus | 3,395 | 235 | 6.9% | 75 | 2.2% | 190 | 5.6% |
| NEO015 | Gastrointestinal cancers - colorectal | 247,885 | 17,000 | 6.9% | 7,080 | 2.9% | 12,685 | 5.1% |
| NEO025 | Skin cancers - melanoma | 25,085 | 1,700 | 6.8% | 795 | 3.2% | 1,240 | 4.9% |
| NEO072 | Neoplasms of unspecified nature or uncertain behavior | 254,540 | 16,955 | 6.7% | 6,780 | 2.7% | 12,315 | 4.8% |
| NEO043 | Urinary system cancers - bladder | 87,280 | 5,780 | 6.6% | 2,330 | 2.7% | 4,470 | 5.1% |
| NEO037 | Female reproductive system cancers - vagina | 2,540 | 165 | 6.5% | 50 | 2.0% | 130 | 5.1% |
| NEO065 | Multiple myeloma | 117,595 | 7,415 | 6.3% | 2,500 | 2.1% | 5,760 | 4.9% |
| NEO010 | Head and neck cancers - all other types | 12,205 | 760 | 6.2% | 210 | 1.7% | 625 | 5.1% |
| NEO064 | Leukemia - all other types | 31,000 | 1,910 | 6.2% | 695 | 2.2% | 1,425 | 4.6% |
| NEO009 | Head and neck cancers - tonsils | 8,185 | 500 | 6.1% | 220 | 2.7% | 355 | 4.3% |
| NEO007 | Head and neck cancers - pharyngeal | 5,355 | 320 | 6.0% | 190 | 3.6% | 170 | 3.2% |
| NEO005 | Head and neck cancers - nasopharyngeal | 3,235 | 190 | 5.9% | 65 | 2.0% | 140 | 4.3% |
| NEO034 | Female reproductive system cancers - fallopian tube | 3,595 | 210 | 5.8% | 80 | 2.2% | 155 | 4.3% |
| NEO027 | Skin cancers - squamous cell carcinoma | 13,220 | 760 | 5.7% | 270 | 2.0% | 600 | 4.5% |
| NEO061 | Leukemia - chronic lymphocytic leukemia (CLL) | 83,155 | 4,775 | 5.7% | 2,035 | 2.5% | 3,380 | 4.1% |
| NEO003 | Head and neck cancers - throat | 7,300 | 415 | 5.7% | 160 | 2.2% | 295 | 4.0% |
| NEO050 | Endocrine system cancers - thyroid | 17,935 | 985 | 5.5% | 410 | 2.3% | 685 | 3.8% |
| NEO039 | Male reproductive system cancers - prostate | 182,295 | 9,960 | 5.5% | 4,260 | 2.3% | 7,325 | 4.0% |
| NEO006 | Head and neck cancers - hypopharyngeal | 3,335 | 180 | 5.4% | 55 | 1.7% | 140 | 4.2% |
| NEO008 | Head and neck cancers - laryngeal | 18,470 | 970 | 5.3% | 335 | 1.8% | 690 | 3.7% |
| NEO004 | Head and neck cancers - salivary gland | 4,755 | 235 | 4.9% | 115 | 2.4% | 165 | 3.5% |
| NEO044 | Urinary system cancers - ureter and renal pelvis | 10,570 | 520 | 4.9% | 150 | 1.4% | 415 | 3.9% |
| NEO068 | Myelodysplastic syndrome (MDS) | 70,155 | 3,360 | 4.8% | 835 | 1.2% | 2,740 | 3.9% |
| NEO026 | Skin cancers - basal cell carcinoma | 10,530 | 500 | 4.7% | 230 | 2.2% | 335 | 3.2% |
| NEO002 | Head and neck cancers - lip and oral cavity | 29,585 | 1,400 | 4.7% | 550 | 1.9% | 1,005 | 3.4% |
| NEO028 | Skin cancers - all other types | 11,760 | 540 | 4.6% | 240 | 2.0% | 380 | 3.2% |
| NEO062 | Leukemia - chronic myeloid leukemia (CML) | 28,935 | 1,160 | 4.0% | 335 | 1.2% | 880 | 3.0% |
| NEO029 | Breast cancer - ductal carcinoma in situ (DCIS) | 6,775 | 235 | 3.5% | 100 | 1.5% | 170 | 2.5% |
| NEO001 | Head and neck cancers - eye | 2,170 | 75 | 3.5% | 40 | 1.8% | 45 | 2.1% |
| NEO055 | Endocrine system cancers - pituitary gland | 250 | 5 | 2.0% | - | 0.0% | 5 | 2.0% |
| VTE: Venous Thromboembolism, PE: Pulmonary Embolism, DVT: Deep Vein Thrombosis | | | | | | | | |

| **Supplementary Table 2: Odds ratio of venous thromboembolism in hospitalizations in patients**  **with or without cancer diagnosis, excluding NEO072 (neoplasms of unspecified nature or uncertain behavior)** | | | | | | | | | | | | | | | |
| --- | --- | --- | --- | --- | --- | --- | --- | --- | --- | --- | --- | --- | --- | --- | --- |
|  | **Thromboembolus Diagnosis** | | **Unadjusted** | | | | **Adjusted** | | | | |  |  |  |  |
|  | **Cancer**  **Present (2,704,073)** | **Cancer Absent (25,738,936)** | **OR^a^** | **CI 95%** | | ***p*** | **aOR^b^** | | **CI 95%** | | ***p*** |  |  |  |  |
| VTE | 221,440 (8.2%) | 900,424 (3.5%) | 2.46 | 2.43-2.49 | | <0.001 | 1.83 | | 1.81-1.85 | | <0.001 |  |  |  |  |
| PE | 91,095 (3.4%) | 424,764 (1.7%) | 2.08 | 2.04-2.11 | | <0.001 | 1.55 | | 1.53-1.58 | | <0.001 |  |  |  |  |
| DVT | 163,300 (6%) | 607,629 (2.4%) | 2.66 | 2.63-2.69 | | <0.001 | 1.96 | | 1.93-1.98 | | <0.001 |  |  |  |  |
| *VTE: Venous Thromboembolism, PE: Pulmonary Embolism, DVT: Deep Vein Thrombosis*  *^a^Unadjusted Odds Ratio (OR) of specified thrombosis with or without a cancer diagnosis*  *^b^Age, gender, comorbidity (hypertension, type 2 diabetes mellitus, tobacco use, obesity, and sepsis),*  *and APRDRG disease severity adjusted Odds Ratio (aOR)* | | | | | | | | | | | | | | |  |
| **Supplementary Table 3: Odds ratio of mortality in cancer-associated hospitalizations with or without**  **venous thromboembolism diagnosis, excluding NEO072 (neoplasms of unspecified nature**  **or uncertain behavior)** | | | | | | | | | | | | | | | |
|  | **All-Cause Mortality** | | **Unadjusted** | | | | | **Adjusted** | | | | |  |  |  |
|  | **With**  **thrombus (221,410)** | **Without thrombus (2,482,203)** | **OR^a^** | | **CI 95%** | **p** | | **aOR^b^** | | **CI 95%** | **p** | |  |  |  |
| VTE | 23,790 (10.7%) | 147,435 (5.9%) | 1.91 | | 1.85-1.97 | <0.001 | | 1.61 | | 1.56-1.67 | <0.001 | |  |  |  |
| PE | 10,915 (12%) | 160,310 (6.1%) | 2.08 | | 1.99-2.18 | <0.001 | | 1.88 | | 1.79-1.97 | <0.001 | |  |  |  |
| DVT | 16,325 (10%) | 154,900 (6.1%) | 1.71 | | 1.65-1.78 | <0.001 | | 1.41 | | 1.36-1.47 | <0.001 | |  |  |  |
| *VTE: Venous Thromboembolism, PE: Pulmonary Embolism, DVT: Deep Vein Thrombosis*  *^a^Unadjusted Odds Ratio (OR) of mortality in hospitalizations with cancer and specified thrombosis diagnoses*  *^b^Age, gender, comorbidity (hypertension, type 2 diabetes mellitus, tobacco use, obesity, sepsis), and*  *metastatic disease adjusted Odds Ratio (aOR)* | | | | | | | | | | | | | |  |  |
